# Cutaneous Manifestations of Non-Celiac Gluten Sensitivity: Clinical Histological and Immunopathological Features

**DOI:** 10.3390/nu7095368

**Published:** 2015-09-15

**Authors:** Veronica Bonciolini, Beatrice Bianchi, Elena Del Bianco, Alice Verdelli, Marzia Caproni

**Affiliations:** 1 Department of Surgery and Translational Medicine, Section of Dermatology, University of Florence, Viale Michelangiolo 41, Florence 50125, Italy; E-Mails: beatrice.bianchi@unifi (B.B.); elena.delbianco@unifi (E.D.B.); alice.verdelli@hotmail (A.V.); 2 Director SOS Skin Immunopathology and Rare Dermatological Diseases Unit, 1st Dermatological Clinic ASF-Piero Palagi, Department of Medical and Surgical Critical Care, Section of Dermatology, University of Florence, Viale Michelangiolo 41, Florence 50125, Italy; E-Mail: marzia.caproni@unifi

**Keywords:** non-celiac gluten sensitivity, cutaneous gluten sensitivity, skin manifestations, direct immunofluorescence

## Abstract

Background: The dermatological manifestations associated with intestinal diseases are becoming more frequent, especially now when new clinical entities, such as Non-Celiac Gluten Sensitivity (NCGS), are identified. The existence of this new entity is still debated. However, many patients with diagnosed NCGS that present intestinal manifestations have skin lesions that need appropriate characterization. Methods: We involved 17 patients affected by NCGS with non-specific cutaneous manifestations who got much better after a gluten free diet. For a histopathological and immunopathological evaluation, two skin samples from each patient and their clinical data were collected. Results: The median age of the 17 enrolled patients affected by NCGS was 36 years and 76% of them were females. On the extensor surfaces of upper and lower limbs in particular, they all presented very itchy dermatological manifestations morphologically similar to eczema, psoriasis or dermatitis herpetiformis. This similarity was also confirmed histologically, but the immunopathological analysis showed the prevalence of deposits of C3 along the dermo-epidermal junction with a microgranular/granular pattern (82%). Conclusions: The exact characterization of new clinical entities such as Cutaneous Gluten Sensitivity and NCGS is an important objective both for diagnostic and therapeutic purposes, since these are patients who actually benefit from a GFD (Gluten Free Diet) and who do not adopt it only for fashion.

## 1. Introduction

The cutaneous manifestations of intestinal bowel diseases have always been an obstacle either for the gastroenterologists or dermatologists. A close collaboration between these practitioners is required because of the wide range of skin diseases that may be associated both with celiac diseases (CD) and the inflammatory bowel diseases (IBD) [1,2,3,4,5]. For a long time, our group has been dedicated to the characterization of specific and non-specific cutaneous manifestations related to bowel diseases, in particular to CD. In a previous article we already described all skin manifestations in patients with celiac disease, adopting the classification of Humbert *et al.* [6], that divided them in two groups: those improved by gluten free diet and those occasionally associated with CD; and in four categories: autoimmune, allergic, inflammatory, and miscellaneous [5].

Recently, the study of Volta *et al.* [7] conducted with the aim to define the clinical picture of this new syndrome and to establish roughly its prevalence compared to CD showed a strong correlation with female gender and adult age. The study reiterated the relationship with the irritable bowel syndrome (IBS). The diagnosis is currently based only on clinical suspicion because of the lack of valid biomarkers and of the gold standard assay dietary elimination of gluten which is followed by a randomized double-blind, placebo-controlled (DBPC) food challenge (even if the last one is difficult to adopt routinely in clinical practice). Confirming our hypothesis of skin involvement, 29% out of 486 patients with suspected Non-Celiac Gluten Sensitivity (NCGS) that took part in the study presented skin rash and 18% of them not well defined dermatitis.

We believe that these cutaneous manifestations should be better defined with a clinical evaluation by expert dermatologists, who may identify specific patterns through finalized immune-histological studies. As it has already happened with CD and dermatitis herpetiformis (DH), also in these patients can be identified a specific skin pattern, which itself may be sufficient to make a diagnosis of bowel disease.

The etiology and the pathophysiology of NCGS is under scrutiny. A lot of people spontaneously adopt a gluten free diet, solving their intestinal and extra-intestinal symptoms such as diarrhea, abdominal discomfort or pain, bloating, flatulence and headache, lethargy, attention-deficit/hyperactivity disorder, ataxia or recurrent oral ulceration. Over the years, the gluten free diet (GFD) has become a fashion, and, due to this, skepticism among some authors in regard to the NCGS has arisen. Nevertheless, scientific papers that describe this new entity are still increasing in number [8], and, in our work experience, skin lesions observed in patients with NCGS are actually sensitive to GFD. In fact, in the Western world, the consumption of wheat that has increased in the last half century, proportionally to the standard of living and life length [9], now has an opposite trend in Europe, Australia, New Zealand and United States of America [10]. However, it is different for the large populations in China and India [11], where currently wheat is more desirable than the rice.

Recently, Hollon J. *et al.* [12] showed that gliadin exposure induced an increase in the intestinal permeability in all individuals, regardless of whether they have CD or not. Their study suggests that the gluten exposure leads to altered barrier function in NCGS patients as well. It results in an exaggerated increase of the intestinal permeability that may confirm the hypothesis of the involvement of an innate immune response to this new syndrome. In respect to this, a US group reported an increased density of CD3 + intra-epithelial T-cells (intra-epithelial lymphocytes IELs) and a higher expression of mucosal Toll-like receptor 2 in NCGS patients compared to controls indicating immune activation in NCGS. The same study showed decreased mucosal level of mRNA for FOXP3 in NCGS patients compared to controls. This can be read as a possible sign of activation of T regulatory cells [13].

At the moment, there are only some hypotheses in regard to the pathogenic mechanisms at the base of this new condition and they still lead to confusion. In part, it is because of their significant impact on the media and socio-economic awareness which has been created. One hypothesis, which is based on what has been demonstrated so far, is that NCGS may be characterized by an activation of innate immunity [13,14], but probably all these symptoms may be the result of an exaggerated response of a normal individual to a meal containing gluten. Indeed, gluten is a component of the more complex protein mixture contained in wheat flour that can cause a significant increase of the intestinal fermentation processes in healthy individuals, through its opioid properties that are naloxone reversible [15]. Moreover, it can induce modifications in the intestinal transit, a possible low degree of intestinal inflammation (in experimental models) [16], and, last but not the least, a *nocebo* effect.

As the previously mentioned association between systemic diseases and cutaneous manifestations is not new, probably this link may be related to the constant communication among the different organ systems of our body.

The purpose of this study is mainly to define the cutaneous manifestations in the course of NCGS and, eventually, to characterize the new pathologic entities known as CGS.

## 2. Experimental Section

In the collaboration with gastroenterologists and physicians, we enrolled consecutive patients affected by NCGS and with non-specific cutaneous manifestations improved by gluten free diet. In accordance with the new algorithm proposed by Sapone A. *et al.* [17], we excluded other gluten reactions as wheat allergy (WA) and CD performing standard screening. Furthermore, specific skin prick tests and wheat specific serum IgE were done in order to exclude WA. Moreover, serological assay with specific antibodies (anti-tissue transglutaminase antibodies (tTG) IgA and IgG, anti-endomysial antibodies (EMA), anti-gliadin antibodies (AGA), deamidated gliadin peptide (DGP) and total IgA) and esophagogastroduodenoscopy with multiple biopsies served to exclude CD. We enrolled NCGS patients only after dietary elimination of gluten followed by DBPC, also useful to define the time of recurrence of the lesions with the reintroduction of gluten in the diet. We used a special form to collect their clinical data (gender, age, duration, family history of skin and/or intestinal diseases, morphology and localization of cutaneous lesions, gastrointestinal symptoms and other symptoms compatible to NCGS) which allowed us to define the clinical features of CGS and its possible variants (Table S1). We also reported time of resolution of the lesions adopting GFD (Table S1), with the purpose to compare the cutaneous manifestations of other gluten-sensitive diseases such as DH and collected skin samples for histological and direct immunofluorescence studies to define any specific patterns of CGS in the future. A written informed consent was obtained from all patients and procedures were carried out in accordance with the ethical standards of the Committee on Human Experimentation of the Department of the Dermatological Science and the Declaration of Helsinki. For the immunopathological study, skin samples from all the patients and controls were collected and they were immediately frozen at −80 °C in liquid nitrogen. The frozen specimens were cut into 5 μm thick sections: two of them were layered on each slide and the slides were stored at −20 °C until being stained. For staining, the sections were warmed to room temperature. Optimally diluted fluorescein isothiocyanate (FITC)—labeled monospecific immunoglobulins (IgG, IgA, IgM, C3 (DakoCytomation, Glostrup, Denmark)) was layered onto the sections and incubated at 37 °C for 45 min–1 h. Then, the sections were washed thrice in PBS (pH 7.2, 0.1 M), mounted in buffered glycerin and finally analyzed under a Nikon C2 confocal microscope (Nikon, Tokyo, Japan) The following features were documented: (a) the nature of the immune deposits: IgG, IgA, IgM, C3; (b) the site of the immune deposits: dermal-epidermal junction (DEJ), intercellular spaces (ICS) or perivascular site; (c) the pattern of the immune deposits: granular, linear or mixed (granular/linear).

## 3. Results

In this prospective study, we enrolled 17 consecutive patients affected by NCGS after having considered gastroenterology and allergy in order to exclude other forms of gluten sensitivity such as CD and WA. We collected their clinical data in Table S1 (gender, age, duration, family history of skin and/or intestinal diseases, morphology and localization of cutaneous lesions, gastrointestinal or other symptoms compatible with NCGS, time of recurrence of the lesions after gluten re-exposure). The patients involved in the study were between 5 and 69 years old with an average age of 36 and most of them were females (Female: 76%; Male: 24%).

There were only 4 pediatric patients, all female. At the time of our observation the majority of patients reported gastrointestinal symptoms of varying intensity similar to IBS, such as abdominal pain, bloating, flatulence, diarrhea or constipation. Some of them, for example the skin manifestations, generally improved with spontaneously adopted GFD. Ten of the enrolled patients (59%) presented cutaneous manifestations for about one year, while in the others there was much longer disease duration with a typical chronic relapsing course.

Morphologically, the lesions were mainly erythematous, excoriated papular-vesicular and extremely itchy, similar to subacute eczema or DH. Nevertheless, some patients had hyperkeratotic scaly lesions instead overlying mild erythematous infiltrative lesions and they were associated with excoriations similar to chronic psoriasis. In order of frequency, the sites of the lesions were: extensor surfaces of upper limbs (elbows and back of the hands (94% and 6%)), extensor surfaces of lower limbs (knees) (59%), bottom (29%), chest (18%), neck (18%), the palms of the hands (6%), extensor surface of upper limbs (6%) and face (6%) (Figures S1–S5).

The mean time of disappearance of the skin lesions after adoption of GFD was one month or so as it is shown in Table S1.

Given the controversial relationship with NCGS, the serological screening for CD included also AGA, but only three of 17 (18%) patients showed positive results.

Histological features are shown schematically in Table S2. It was possible to outline a specific histological pattern, but it is important to point out the similarity with psoriasis, eczema and DH.

Finally, in Table S3, we reported the immunopathological features obtained by a direct immunofluorescence (DIF) test on a skin biopsy got from a perilesional skin, as it is usual in suspected DH to preserve immunological deposits. We described the type of reactant that forms the immunological deposit (IgG, IgM, IgA, C3 or C1q) and the site (DEJ—Dermoepidermal junction, PV—Perivascular, FB—Fluorescent bodies). The patterns of distribution along the DEJ were microgranular/granular in all patients. For each reactant we calculated (IgG, IgM, IgA, C3 and C1q) in how many patients it appears in different sites (DEJ, PV, FB). This is presented in percentages in Table S3. The C3 fraction of the complement system is the only reactant percentage in more than a half of the patients (82%) localized in particular along the DEJ (Figure S6).

## 4. Discussion

We are aware of the limitations of this study arising from, the limited number of patients enrolled, but we used very tight exclusion criteria, as we did not include patients with an uncertain diagnosis of NCGS.

The common clinical feature of all the patients involved in this study, as well as the one detached from our clinical experience, was the severe itching. It was difficult to treat with standard topical and systemic therapies, but it showed prompt resolution when GFD was introduced: the reaction was faster than in DH patients. Morphologically, the lesions were polymorphic, in terms of development. In fact, initially they were mainly erythematous and papulo-vesicular like eczema and DH, then later, maybe due to a constant scratching, they appeared psoriatic-like. Furthermore, similar to DH, the lesions were more frequently localized on the extensor surfaces of the limbs, in particular on the elbows (94%), followed by knees (59%), than bottom (29%), chest (18%), neck (18%), the palms and back of the hands (6%), extensor surface of upper limbs (6%) and face (6%).

The mean time of disappearance of the skin lesions after adoption of GFD was about one month in the patients enrolled, much shorter than in DH.

On the contrary to DH, we have not identified a specific histological pattern: the histological characteristics may change during the time just as we said previously about the morphology of the lesions. In particular, in the early stages, lymphocytic infiltrate and spongiosis may be present, while in the later phases hyperkeratosis and mixed infiltrate are prevalent. However, to prove this as certain, we need further research with a larger number of patients.

DH IgA deposits along DEJ with typical reinforcing of the dermal papillae represents either the specific immunological pattern or the gold standard for the diagnosis. This is the reason why we excluded DH in all patients enrolled. In fact, the C3 fraction of the complementary system was the only reactant present in more than a half of the patients (82%) in particular localized along the DEJ in granular or micro-granular pattern, while IgA were present in an insignificant number of patients and still without a specific pattern of distribution. Further studies are needed to define the specificity and sensitivity of the C3 deposits along the DEJ in the diagnosis of CGS. If our partial data should be confirmed, the absence of a biomarker for the diagnosis of NCGS may offset the skin biopsy, at least in those patients with cutaneous manifestations, such as in celiac patients with DH [18]. Furthermore, the involvement of the complementary system may confirm the predominant role of an innate immunity in the pathogenesis of CGS, as suggested for NCGS and the attempt to induce an antigen-specific tolerance resulted in the production of transforming growth factor-beta and interleukin-10 [19].

Even if in our research we have not considered a control after the GFD, all enrolled patients are still in follow-up and they are showing rapid clinical and immunopathological resolution with a disappearance of lesions and deposits only after a month of GFD.

To sum up, in our experience a lot of non-celiac patients with intestinal symptoms compatible with NCGS showed non-specific, often itchy, dermatoses that, in some of the cases, were overlapped by morphology and localization with the DH, the specific cutaneous manifestation of CD. In other patients, instead, it coincided with the eczematoid or psoriasiform appearance. Moreover, if a gluten free diet is adopted to address the intestinal disorders, skin manifestations are resolved or they improve significantly. The histological and immunopathological assays performed on skin samples exclude specific skin diseases of CD and the allergy skin tests exclude sensitization to gluten. Therefore, it is reasonable to assume that there may also appear skin manifestations among the extraintestinal manifestations of NCGS or that “cutaneous gluten sensitivity” (CGS) exists and needs to be characterized and this is the aim of this study. Probably, the late diagnosis in patients we evaluated was due to the lack of knowledge of this new clinical entity and its potential connections with other systems, primarily the skin.

## 5. Conclusions

At the moment, the results of our study do not allow the exact characterization of a new skin disease related to NCGS. The skin lesions observed were similar both to eczema and psoriasis and did not show a specific histological pattern. Furthermore, no serological marker was useful to identify these patients. The only data common to most of these patients affected by NCGS associated to non-specific skin manifestations are:

1. the itching;
2. the presence of C3 at the dermoepidermal junction;
3. a rapid resolution of lesions when adopting the gluten free diet.

Nevertheless, we want to stress once again the importance of a close collaboration between gastroenterologists and dermatologists, because the gastrointestinal system and the skin may be considered more and more “two sides of the same coin”. The exact characterization of new clinical entities such as CGS and NCGS is an important objective both for diagnostic and therapeutic purposes, since these are patients who actually benefit from a GFD and who do not adopt it only for fashion. Therefore, dermatologists must be familiar with the cutaneous manifestations and symptoms of gastrointestinal disorders. An appropriate understanding, work-up, consultation and management will help to identify the important cutaneous—gastrointestinal connection and ensuring that this important gastroenterological disease in patients with skin manifestations is not ignored.

Finally, we suggest an accurate follow-up of all patients who report intense itching and gastrointestinal disorders, even when histology and morphology of the skin lesions do not identify a specific skin disease. We also suggest the adoption of GFD for at least three months assessing any positive effects.

## Supplementary Files

Supplementary File 1
