# Supplementary material for: Cutaneous Manifestations of Non-Celiac Gluten Sensitivity: Clinical Histological and Immunopathological Features
Source: Nutrients. 2015 Sep 15;7(9):7798–805. doi: 10.3390/nu7095368 (PMC4586563; doi:10.3390/nu7095368)
Supplement: Supplementary File 1 [file nutrients-07-05368-s001.docx]

**Supplementary Materials**

**Table S1.** Clinical features of enrolled patients.

| **Patient** | **Gender** | **Age** | **Duration of skin manifestations (year)** | **Family history of skin and/or intestinal diseases** | **Gastrointestinal symptoms** | **Skin manifestations (morphology and localization)** |
| --- | --- | --- | --- | --- | --- | --- |
| 1 | Female | 5 | 1 | CD, DE and Brugada syndrome | None | Itching associated with excoriated lesions on elbows and knees |
| 2 | Female | 11 | 3 | CD, DE and Brugada syndrome | Abdominal pain and bloating | Itching associated with excoriated lesions on elbows and knees |
| 3 | Female | 7 | 3 | CD, DE and Brugada syndrome | None | Itching associated with papular-vesicular excoriated lesions on elbows and knees |
| 4 | Female | 69 | 6 | None | Abdominal pain and bloating | Itching associated with erythematous excoriated lesions on elbows, knees and chest |
| 5 | Female | 29 | 1 | None | Abdominal pain, constipation and diarrhea | Itching associated with papulopustolar excoriated lesions on elbows and neck |
| 6 | Female | 27 | 1 | None | Abdominal pain | Itching associated with papular-vesicular excoriated lesions on elbows and knees |
| 7 | Female | 50 | 1 | CD | Abdominal pain, bloating and diarrhea | Itching associated with erythematous scaly lesions on elbows and knees |
| 8 | Female | 34 | 10 | None | None | Itching associated with papular-vesicular excoriated lesions on backs and palms of hands |
| 9 | Male | 30 | 1 | None | Abdominal pain and bloating | Itching papular lesions on elbows |
| 10 | Female | 58 | 1 | None | Flatulence, diarrhea and constipation | Itching grouped  papulo-vesicular lesions on neck and elbows |

**Table S1.** *Cont.*

| **Patient** | **Gender** | **Age** | **Duration of skin manifestations (year)** | **Family history of skin and/or intestinal diseases** | **Gastrointestinal symptoms** | **Skin manifestations (morphology and localization)** |
| --- | --- | --- | --- | --- | --- | --- |
| 11 | Female | 16 | 1 | None | Abdominal pain and bloating | Itching erythematous excoriated lesions on bottom, chest, extensor surfaces of upper and lower limbs |
| 12 | Male | 49 | 2 | None | Abdominal pain | Itching papular-vesicular grouped lesions on elbows, back of wrists and bottom |
| 13 | Female | 40 | 1 | None | Abdominal pain and flatulence | Itching papular-erythematous lesions on extensor surfaces of upper and lower limbs, face and neck |
| 14 | Female | 50 | 1 | None | None | Itching erythematous escoriated lesions of extensor surfaces of upper and lower limbs and bottom |
| 15 | Male | 38 | 1 | None | None | Itching papular-erythematous lesions on elbows and bottom |
| 16 | Female | 62 | 1 | None | None | Itching papular-erythematous lesions on elbows and bottom |
| 17 | Male | 43 | 2 | None | Abdominal pain, bloating, flatulence | Itching erythematous- papular-vesicular lesions on chest, extensor surfaces of upper and lower limbs. |

**Figure S1.** Erythematous-papular lesions on knee.

**Figure S2.** Erythematous papular-vesicular lesions on the chest.

**Figure S3.** Erythematous papular scaly lesions on the elbows.

**Figure S4.** Erythematous grouped popular-vesicular lesions with excoriations on the bottom

**Figure S5.** Erythematous scaly lesions on the back on the hand.

**Table S2.** Histological features of the lesions.

| **Patient** | **Hyperortoparakeratosis** | **Spongiosis** | **Acantosis** | **Dermoepidermal split** | **Lymphocytic infiltrate** | **Granulocyte infiltrated**  **(type)** |
| --- | --- | --- | --- | --- | --- | --- |
| 1 | X | X |  |  | X |  |
| 2 | X | X |  |  | X |  |
| 3 | X |  | X |  | X |  |
| 4 | X |  | X |  | X |  |
| 5 |  |  |  | X | X | X  (neutrophils) |
| 6 |  |  |  |  | X |  |
| 7 |  |  |  |  | X |  |
| 8 |  | X |  |  |  | X (eosinophils) |
| 9 | X | X |  |  | X |  |
| 10 |  |  |  |  | X |  |
| 11 | X |  | X |  | X |  |
| 12 |  |  |  |  |  | X (neutrophils) |
| 13 | X |  |  |  | X |  |
| 14 | X |  |  |  | X | X (eosinophils) |
| 15 |  |  |  |  | X |  |
| 16 | X | X |  |  |  |  |
| 17 |  |  |  | X | X | X |

**Table S3.** Percentage of patients which present the reactants (IgG, IgM, IgA, C3, C1q) in different sites (DEJ, PV, FB).

|  | **IgG** | **IgM** | **IgA** | **C3** | **C1q** |
| --- | --- | --- | --- | --- | --- |
| DEJ | 0% | 41.2% | 11.8% | 82.3% | 29.4% |
| PV | 0% | 5.9% | 11.8% | 11.8% | 11.8% |
| FB | 0% | 35.3% | 23.5% | 11.8% | 0 |


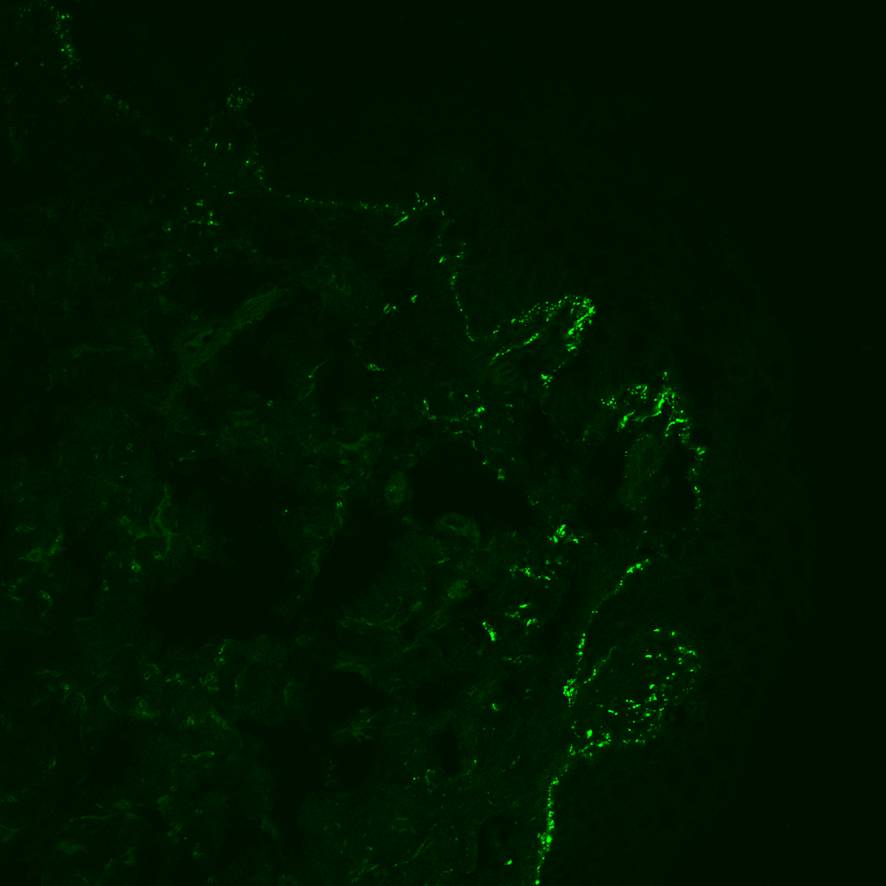


**Figure S6.** Microgranular C3 deposits along the dermo-epidermal junction.

© 2015 by the authors; licensee MDPI, Basel, Switzerland. This article is an open access article distributed under the terms and conditions of the Creative Commons Attribution license (http://creativecommons.org/licenses/by/4.0/).
